# An adaptable toolkit to assess commercial fishery costs and benefits related to marine protected area network design

**DOI:** 10.12688/f1000research.7312.2

**Published:** 2017-02-22

**Authors:** Rémi M. Daigle, Cristián J. Monaco, Ashley K. Elgin

**Affiliations:** 1 Department of Ecology and Evolutionary Biology, University of Toronto, Toronto, Ontario, M5S 3B2, Canada; 2 Department of Zoology and Entomology, Rhodes University, Grahamstown, 6140, South Africa; 3 National Oceanic and Atmospheric Administration, Great Lakes Environmental Research Laboratory, Muskegon, Michigan, 49441, USA

**Keywords:** Cost-benefit analysis, Atlantic Cod, individual based models, Conservation, Fisheries management

## Abstract

Around the world, governments are establishing Marine Protected Area (MPA) networks to meet their commitments to the United Nations Convention on Biological Diversity. MPAs are often used in an effort to conserve biodiversity and manage fisheries stocks. However, their efficacy and effect on fisheries yields remain unclear. We conducted a case-study on the economic impact of different MPA network design strategies on the Atlantic cod ( *Gadus morhua*) fisheries in Canada. The open-source R package that we developed to analyze this case study can be customized to conduct similar analyses for other systems. We used a spatially-explicit individual-based model of population growth and dispersal coupled with a fisheries management and harvesting component. We found that MPA networks that both protect the target species’ habitat and were spatially optimized to improve population connectivity had the highest net present value (i.e., were most profitable for the fishing industry). These higher profits were achieved primarily by reducing the distance travelled for fishing and reducing the probability of a moratorium event. These findings add to a growing body of knowledge demonstrating the importance of incorporating population connectivity in the MPA planning process, as well as the ability of this R package to explore ecological and economic consequences of alternative MPA network designs.

## Introduction

Marine Protected Areas (MPAs) have risen to be among the most popular measures to conserve biodiversity and manage populations subjected to strong fishing pressure. MPAs offer ‘safe zones’ for individuals to breed and grow, thus potentially facilitating population persistence. Biological impacts of MPAs also have been shown to extend beyond their boundaries via “spillover effects”, potentially benefitting adjacent fisheries and thus contributing to existing management strategies ( Bellier *et al.*, 2013; Kellner *et al.*, 2007; Sanchirico *et al.*, 2006). Concomitant with the global establishment of MPAs, there has been a growing field of science seeking to objectively quantify the contribution of MPAs to the conservation of commercially and culturally important species. Importantly, studies are highlighting the need for improvements on design ( Gaines *et al.*, 2010). Careful consideration should be paid to the placement of MPAs relative to the natural history of focal species, particularly if connectivity is rooted in the objectives and/or design. For example, studies based on genetic analyses have revealed that invertebrate larvae disperse shorter distances than fish species (~50–100 vs. 100–200 km, respectively) ( Kinlan & Gaines, 2003; Shanks *et al.*, 2003), suggesting that optimal size and spacing between MPAs should be adjusted according to the target species.

Dispersal processes are particularly relevant to MPA design because many marine species under protection by MPAs disperse during different life stages. The dynamics and persistence of populations are influenced by connectivity, which in turn is regulated by dispersal during the larval, juvenile or adult phases ( Cowen & Sponaugle, 2009; Grüss *et al.*, 2011; Levin, 2006). Environmental variables (e.g. temperature, currents) may also influence these processes. For instance, the temperature dependence of the planktonic larval duration (PLD) may add a latitude-dependent element to dispersion, which is rarely incorporated into MPA design protocols because of a scarcity of dispersal data ( Laurel & Bradbury, 2006).

 A promising effort to account for environmental complexities has been the development of MPA networks; that is, a series of independent protected areas interconnected by the movement of organisms between them ( Avasthi, 2005; Gaines *et al.*, 2010). For these networks to succeed, managers are required to make decisions about the optimal size, location, and number of MPAs to implement ( Halpern, 2003). While poorly chosen locations for reserves may have negative effects on populations’ productivity ( Costello & Polasky, 2008; Crowder *et al.*, 2000), well-designed networks, with highly connected reserves, may allow for increased reproduction and survival, although much of the evidence is theoretical or indirect.

Currently, the theoretical importance of connectivity is well established in the ecological literature (e.g., Gaines *et al.*, 2010; Palumbi, 2004). Although one of the four main design principles for MPAs is connectivity ( UNEP-CBD, 2011), many networks of MPAs have not adequately considered this factor in their designs ( Allison *et al.*, 2003; Hastings & Botsford, 2003; Lester *et al.*, 2009). Globally, only 18 to 49% of MPAs are regarded as part of a connected network depending on which definition of “connected network” is used ( Wood *et al.*, 2008). These definitions range from having at least one other MPA greater than 12.5 km ^2^ within 10–20 km to at least one other MPA greater than 3.14 km ^2^ within 20–150 km. There is also a bias towards large reserves, as the ten largest MPAs (most of which were created after 2005) comprise 53% of the protected area ( Devillers *et al.*, 2015). As of 2011, California is the only jurisdiction that uses both size and spacing in MPA design ( Moffitt *et al.*, 2011). Additionally, there is a spatial bias in protection due to jurisdictional, political and logistical concerns. The pelagic environment, which constitutes 99% of biosphere volume and supplies >80% of human fish food supply, may be <0.1% protected ( Game *et al.*, 2009) while areas within the exclusive economic zones are 1.5% protected ( Wood *et al.*, 2008). However, these estimates are now outdated and the area protected is growing every year. While opinions vary regarding the total proportion of area that should be protected (10–30%), it is clear that more MPAs are needed to meet that goal ( Game *et al.*, 2009; Wood *et al.*, 2008). Adding new MPAs is an opportunity to improve the connectivity of existing networks.

MPA research has also revealed that, in addition to the biological and ecological benefits, the success of MPAs needs to be quantified in terms of economic factors. For instance, evidence shows that substantial investment (i.e., USD 5–19 billion; Balmford *et al.*, 2004) would greatly increase the sustainability in the global marine fish catch. In this regard, studies that address the feedbacks between empirical fishery data and human behavior are expected to improve our ability to forecast short-term fisheries ( White *et al.*, 2013). One economic tool that is used to assess fisheries is cost-benefit analysis, which quantifies the balance between costs and benefits of a management action (e.g., Zimmerman *et al.*, 2015). A major challenge for cost-benefit analysis for MPAs is that the costs are incurred immediately, yet the benefits may not be fully realized until the distant future. Hence, it is necessary to conduct cost-benefit analysis over a multi-year time horizon and apply a social discount factor to account for the fact that costs and benefits are valued less in the future than in the present ( Arrow *et al.*, 2013).

Despite the need for more comprehensive analytical models that encompass both biological and economic criteria, few have been developed thus far ( White *et al.*, 2013). One such program is the freeware Marxan, which has been mainly used for conservation planning purposes ( Ball *et al.*, 2009). There are also add-on programs, such as MultCSync ( Moffett *et al.*, 2005) and NatureServe Vista ( http://www.natureserve.org/vista) that can be used to evaluate conservation goals in light of social and economic criteria. However, these tools do not incorporate population connectivity into their analyses by default. Here we introduce a flexible and user-friendly toolkit developed in R as an open-source package for stakeholders to explore the effects of varying different biological and economical parameters on the performance of an MPA network. It addresses specifically the interaction between MPA network design and population connectivity. To illustrate how this toolkit can be applied to different scenarios, we address two specific objectives: (1) examine if having a network of connected MPAs provides more resilience/population stability compared to having a single small, isolated MPA; and (2) determine if there is an economic benefit associated with connected networks of MPAs compared to other potential MPA designs.

We used the Canadian Atlantic cod ( *Gadus morhua*) fishery for our case study because cod is a species with high commercial and cultural value. Cod populations found off the continental shelf of Newfoundland were once one of the world’s richest fishing resources, and shaped Newfoundland society for centuries ( Hamilton & Butler, 2001). Atlantic cod stocks suffered greatly between 1960 and 1990, mostly due to poorly managed fishing pressure. While northwestern cod annual landings were <300 kt before the 1960s, the introduction of advanced fishing gear (e.g. factory trawlers) boosted catches to >800 kt by 1968. The high fishing pressure dramatically reduced standing stocks, and by 1977 fish landings had dropped significantly. Populations seemed to recover in the 1980s, along with increased fishing efforts, which corresponded with a twofold decline in survival probability ( Hutchings & Myers, 1994). As a result, the stocks off Newfoundland and Labrador went commercially extinct (i.e., were no longer economically viable to harvest) in 1992 ( Hutchings & Myers, 1994). The fishery has since shifted to one focused on lobster ($19M), snow crab ($121M), and northern shrimp ($179M) and the total value of the fisheries harvest is now worth more than it was before the moratorium ( Schrank & Roy, 2013). While some cod stocks have started showing tenuous signs of recovery, they are nowhere near pre-moratorium levels ( DFO, 2014); however, protecting a few populations from targeted or bycatch fisheries mortality may improve the probability of recovery. For this reason, we think that exploring the potential benefit of MPA networks for Atlantic Cod would be an informative exercise with implications for management.

## Methods

We developed an individual-based population model for Atlantic Cod ( *Gadus morhua*), which includes fisheries harvesting and alternative management strategies. The model is available as an open-source R package ( Daigle *et al.*, 2017a), which can be downloaded and adapted to the researcher’s specific questions. We describe the general structure of the model below without going into exhaustive detail. Please refer to the toolkit’s ‘ read me’ section for more information on the model mechanics and specific parameter information. The model output presented below is available on zenodo ( Daigle *et al.*, 2017b).

### Model framework

**Figure 1.**
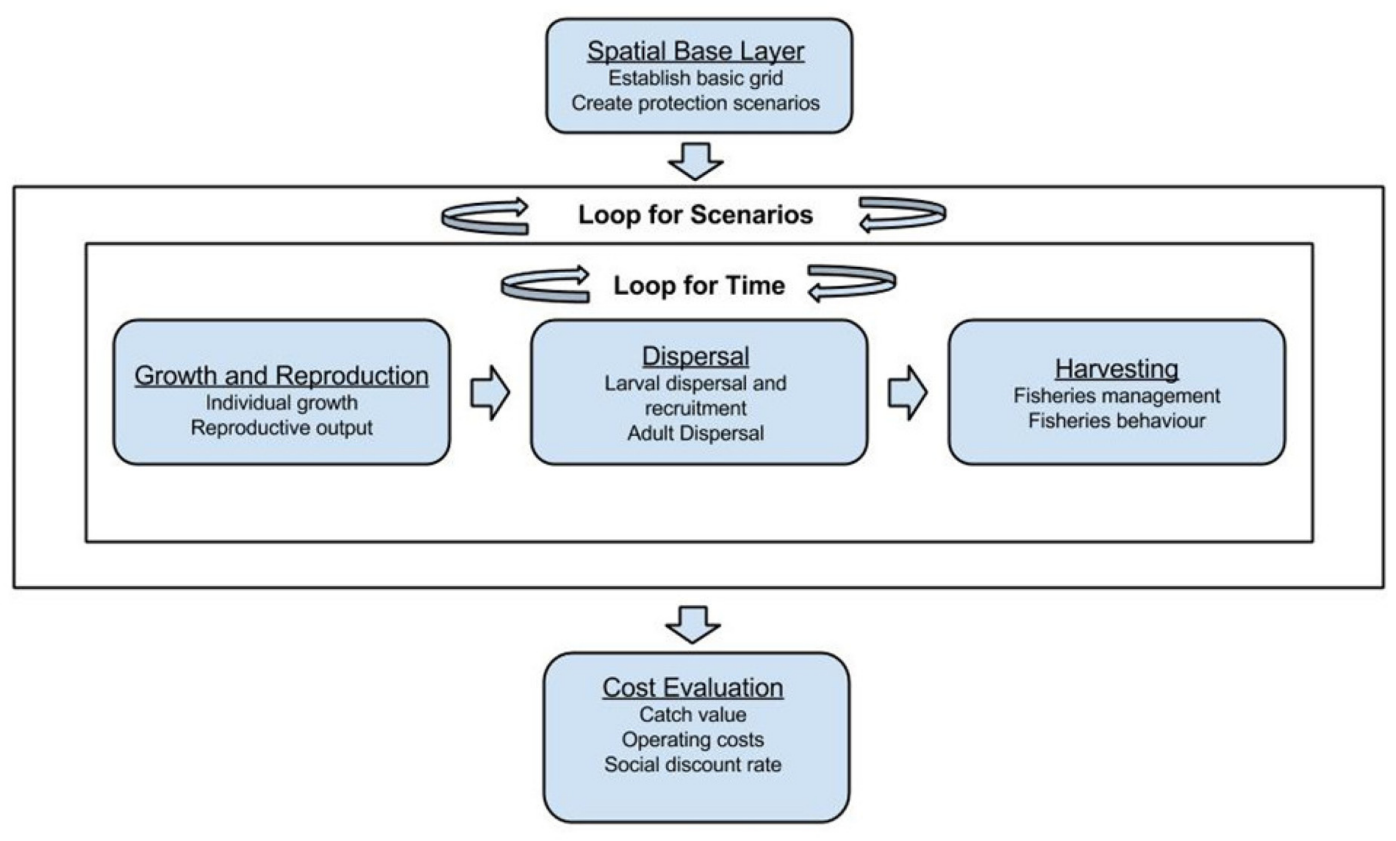
Conceptual diagram of the individual-based population model for Atlantic Cod ( *Gadus morhua*), which includes fisheries harvesting and management. This model evaluates contrasting management scenarios and compares them based on socially discounted net benefits.

**Figure 2.**
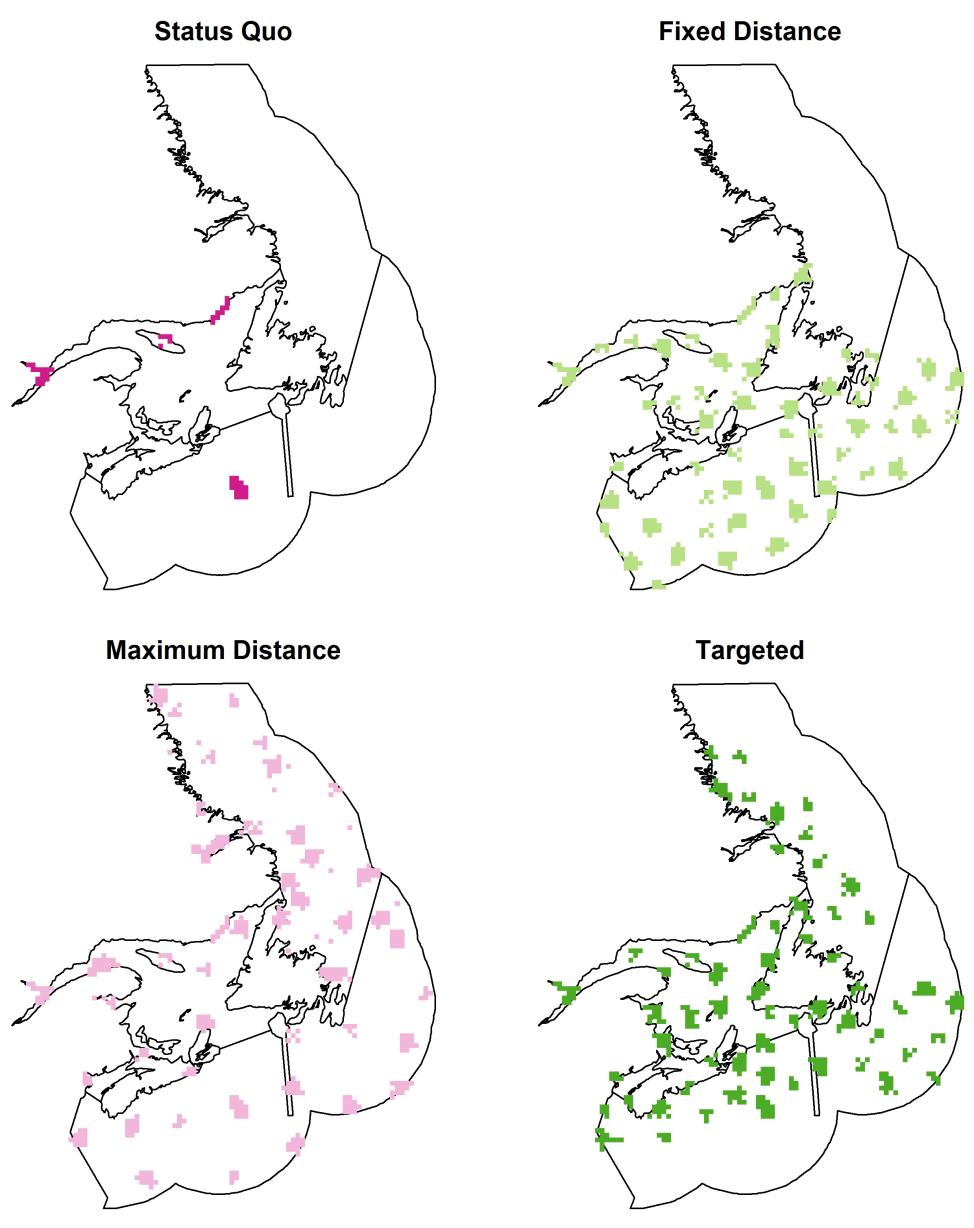
Map of eastern Canadian EEZ (Exclusive Economic Zone) and examples of different scenarios for the planning of Marine Protected Areas (MPAs). The “Status Quo” scenario has all existing MPAs ≥ 400 km ^2^. The MPAs in the “Maximum Distance” scenario have been placed to maximize the distance between them. The MPAs in the “Fixed Distance” scenario have been placed to optimize the distance between them (~75km). The cod’s breeding locations in the “Targeted” scenarios have been protected by default and optimal distance MPAs have been added exclusively in habitat suitable for cod. Scenarios from replicate 1 used as an example, other replicates are available in the appendix ( Figure A1– Figure A99).

 The model is organized hierarchically into modules and sub-modules ( Figure 1) for users to manipulate according to their own requirements. Here we used this model to evaluate four management strategies ( Figure 2) in terms of socially discounted net financial benefit.

The first module establishes a ‘Spatial Base Layer’ by defining a basic grid and creating specific protection scenarios for the resource. The model then runs three modules in sequence; ‘Growth and Reproduction’, ‘Dispersal’, and ‘Harvesting’, all of which determine the population dynamics of the resource (see section *Population dynamics*). These modules are contained in two nested recursive loops, an outer loop that instructs for specific management strategies scenarios (see section *Management strategies scenarios*), and an inner loop that defines the time dimension. Here we ran the model between the years 2000 and 2050, with 1 yr time steps. Finally, the model runs through a ‘Cost of Evaluation’ module, which factors in costs associated with complying with MPA spatial restrictions, the benefits based on catch values, and social discount rates ( Figure 1).

### Population dynamics

The length and weight of fish is estimated from the Von Bertalanffy growth model in ( Knickle & Rose, 2013). It is known that sexual maturity for coastal cod occurs between 2–4 years and 6–9 years for some oceanic stock ( Otterå, 2004); our model approximates this by determining sexual maturity based on a sigmoid logistic curve. The modelled fish begin maturing at 2 y, 50% are mature at 4 y and all are mature at 6 y. Egg production is determined by the weight of spawning females (0.5 million eggs per kg of female). Larval dispersal was approximated with a random walk of 2 cm s ^-1^ over 90 d, which is equivalent to the mean current velocity ( Brander & Hurley, 1992; Otterå, 2004). Adult dispersal was also approximated using a random walk, which was calibrated with tagging data from Lawson & Rose (2000). Our model has four sources of mortality: larval, recruitment, adult, and fisheries. In the model, larval and adult mortality vary randomly through time and space, while recruitment, carrying capacity, and fisheries mortality are linked to adult biomass. Larval and adult mortality are estimated yearly from a beta (α=1000 and β=1.2, which approximates mean larval mortality of 99.88% with a range of 98.98–99.99%; Mountain *et al.*, 2008) and normal distribution (μ = 0.5938 and SD = 0.0517; Swain & Chouinard, 2008), respectively. Recruitment mortality is estimated using a Beverton-Holt model and carrying capacity is assumed to be 0.43 (±0.38 SD) t km ^-2^, which represents an average value for Canadian cod stocks ( Myers *et al.*, 2001). Carrying capacity is also enforced for adult fish and areas with biomass which exceed the carrying capacity are subject to increased mortality. Fisheries mortality is determined by estimating cod biomass by sampling the fish population under 0.1% of the entire EEZ (Exclusive Economic Zone) and using an FMSY (fishing mortality that produces a maximum sustainable yield) of 0.28 ( Mountain *et al.*, 2008). The model sets the target quota to ⅔ of FMSY according to the precautionary principle. Fisherman are assumed to have near perfect knowledge of the optimal fishing locations, and will travel the smallest distance to catch the most fish. We assume that fish under 38 cm are not targeted or caught by fisherman ( Feekings *et al.*, 2013). Fish were valued at a landed price of CAD$1.24 kg ^-1^ ( DFO, 2015). We used operating costs from the Mixed Fleet Fishery ( DFO, 2015) because cod-specific operating costs are difficult to obtain given that cod is caught as bycatch, but not targeted ( Schrank & Roy, 2013).

### Management strategies scenarios

The “Status Quo” scenario has all existing MPAs in Eastern Canada that are at least as large as model cell size (cells are 400 km ^2^). We calculated the profitability ratio using the most recently available estimates of operating costs and landed value for the Canadian Atlantic Region “mixed fleet” fishery ( DFO, 2007). The resulting ratio (landed value/operating cost; ~1.6) indicates the average return on investment in operation for the Status Quo scenario. To estimate operating cost for the other scenarios, we identified distance variable operating costs (fuel and labour), determined the average distance traveled under the Status Quo scenario, and then calculated a distance correction factor based on the difference in distance traveled for a scenario relative to the Status Quo.

The scenarios with MPA protection had 10% of the EEZ closed to fishing, but the criteria for MPA placement was different for each. The MPAs in the “Maximum Distance” scenario have been placed to maximize the distance between them, representing a worst-case scenario in terms of population connectivity. The MPAs in the “Fixed Distance” scenario have been placed to optimize the distance between them (~75km) relative to a mean adult dispersal distance ( Lawson & Rose, 2000). In the “Targeted” scenario, the cod’s breeding locations have been protected by default and optimal distance MPAs have been added exclusively in habitat suitable for cod. The size distribution of the newly generated MPAs were based on that of the coastal and marine protected areas in the World Database on Protected Areas ( UNEP-WCMC, 2015) because this distribution represents what managers have been able to feasibly implement.

### Cost benefit analysis

We calculated net benefits for years 2021–2071 of the model simulation. Social discount rate (SDR) is used to translate future values into present values. The outcome of cost benefit analysis can be influenced by discount rate ( Lupi *et al.*, 2003), so we used a range of values (1.5%, 3.0%, and 6.0%) to test this sensitivity. Discount factor *β _t_* was calculated from *SDR*:

$$\beta_{t}=\frac{1}{{\left(1+SDR\right)}^{t}}$$

Net present value ( *NPV*) was calculated from the sum of present values of net revenue (total revenue from catch *R _t_* minus total operational costs *C _t_*) for each year in the simulation:

$$NPV_{scenario}=\sum_{t=1}^{t=50}(R_{t}-C_{t})\times \beta_{t}$$

### Model output comparisons

To examine the relative effects of scenario on the cumulative present catch value, we calculated three error statistics (mean absolute error, root-mean square error, and mean absolute percent error) using the status quo scenario as a reference. We favored these error metrics to assess magnitude of difference between our model outputs, instead of traditional ANOVA tests, because the large number of replication (100) would have likely influenced the significance level in the latter ( White *et al.*, 2014).

### Sensitivity analysis

We modified select parameters by ± 10% (or ± 1 y for integer values) for the Targeted and Status Quo scenarios. To quantify model sensitivity, we then compared the mean (n = 25) final net present values for the full 50-year time horizon to those of the full models (n = 100). We used the social discount rate of 0.015 because this is the value for which differences among the scenarios are most pronounced.

## Results

**Figure 3.**
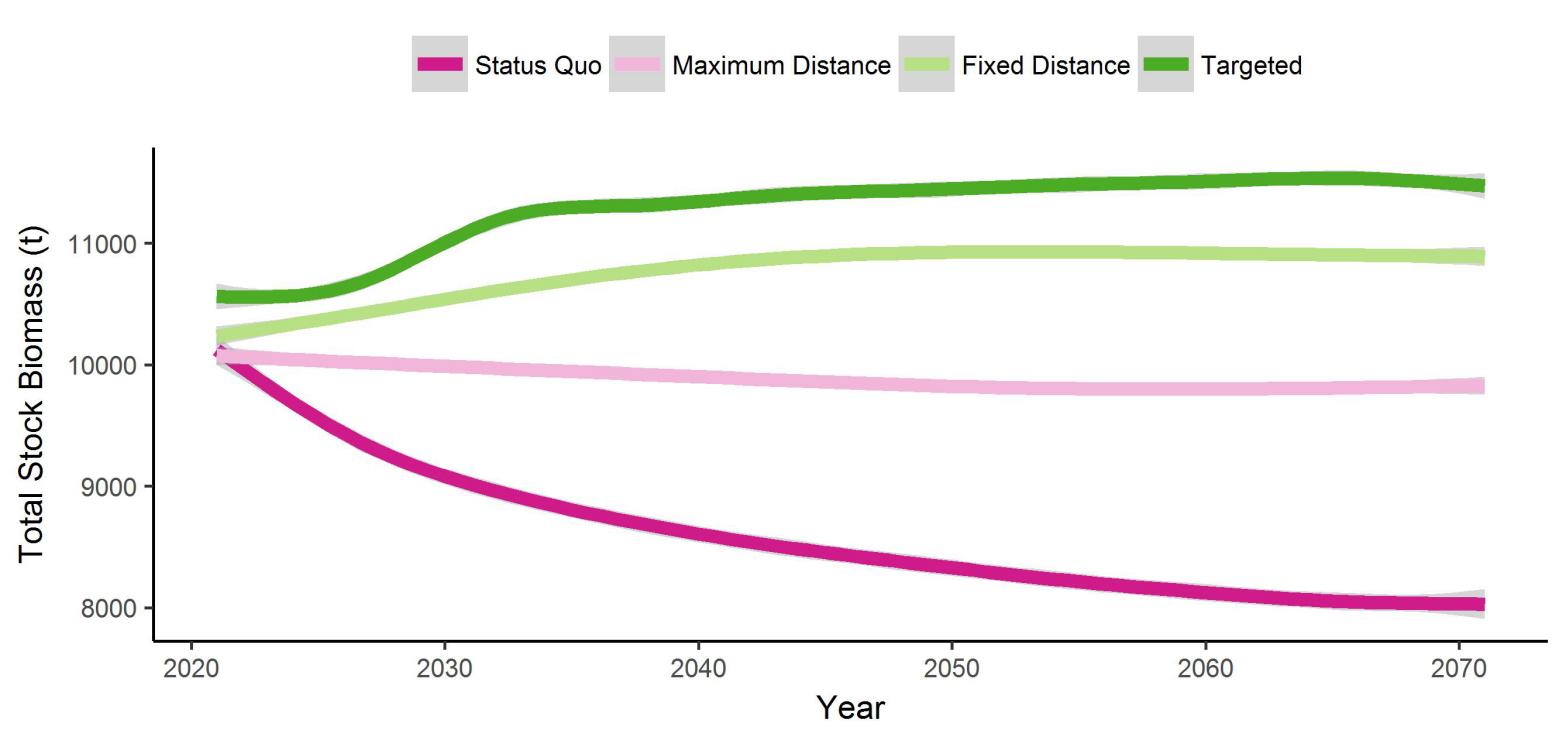
Total stock biomass for each scenario over time. The solid lines represent the mean of all replicates (n=100) and the shaded regions show the standard error around each mean.

**Figure 4.**
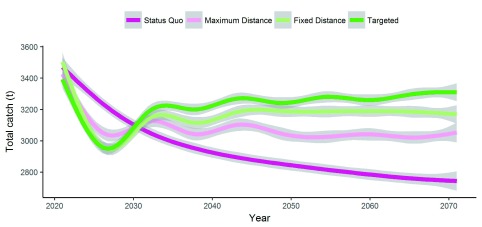
Total catch biomass for each scenario over time. The solid lines represent the mean of all replicates (n=100) and the shaded regions show the standard error around each mean.

Cod biomass did not recover to historically high abundances in any of the four management scenarios. Cod biomass ranged from an average of 8.9 to 11.3 kt depending on the management scenario ( Table 1, Figure 3). While there were no dramatic increases in biomass over time, there is a slight increase in mean biomass for the Fixed Distance and Targeted scenarios. Similarly, the pattern for the harvest follows that of biomass with a few differences. The total catch in all protected scenarios, particularly the Targeted scenario, decreases drastically within the first 5 y, then recovers within ~12 y. In Contrast, total catch in the Status Quo scenario steadily decreases over the entire 51 y. ( Table 1, Figure 4). The probability of a moratorium (triggered when total biomass is below 10 kt) being enforced in a given year was substantially higher for the Status Quo scenario (5.02%) and was lowest for the Targeted scenario (0.98) ( Table1).

**Table 1.** Mean (±SE) summary statistics under each protection scenario for all 51 years and 100 replicates (n=5100). The Biomass is the sum of all the live cod weight in the model domain and catch is the weight of those which were harvested by the fishermen that year. The distance is the weighted mean distance travelled by fishermen (weighted by catch). moratorium (triggered when total biomass is below 10 kt).

##	scenario	Biomass	Catch	Distance	Moratorium
## 1	Status Quo	8597 (± 16)	2938 (± 8)	327 (± 1)	5.02 (± 0)
## 2	Maximum Distance	9887 (± 12)	3074 (± 6)	319 (± 0)	1.37 (± 0)
## 3	Fixed Distance	10768 (± 12)	3161 (± 6)	314 (± 0)	1.12 (± 0)
## 4	Targeted	11282 (± 12)	3224 (± 6)	316 (± 0)	0.98 (± 0)

**Figure 5.**
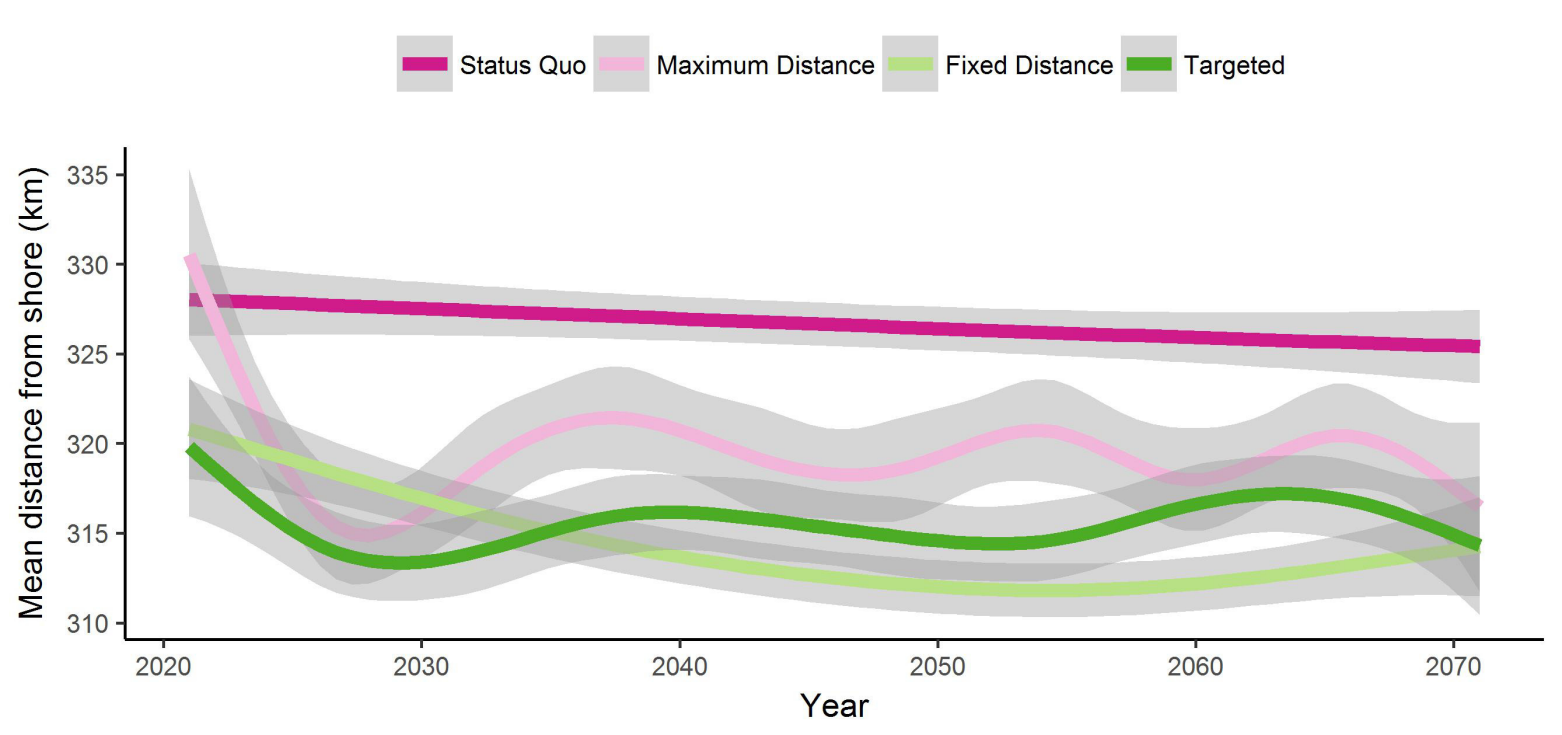
Mean distance from shore of each fish caught for each scenario over time. The solid lines represent the mean of all replicates (n=100) and the shaded regions show the standard error around each mean.

The mean distance from shore of each fish caught was actually highest in the Status Quo scenario ( Table 1, Figure 5). There is a 5 to 10 yr period of adjustment to the management scenario regulation, but the distances in each scenario achieve a stable plateau. On average, fish were caught 327 km from shore in the Status Quo scenario while only 314 km in the Targeted scenario.

**Figure 6.**
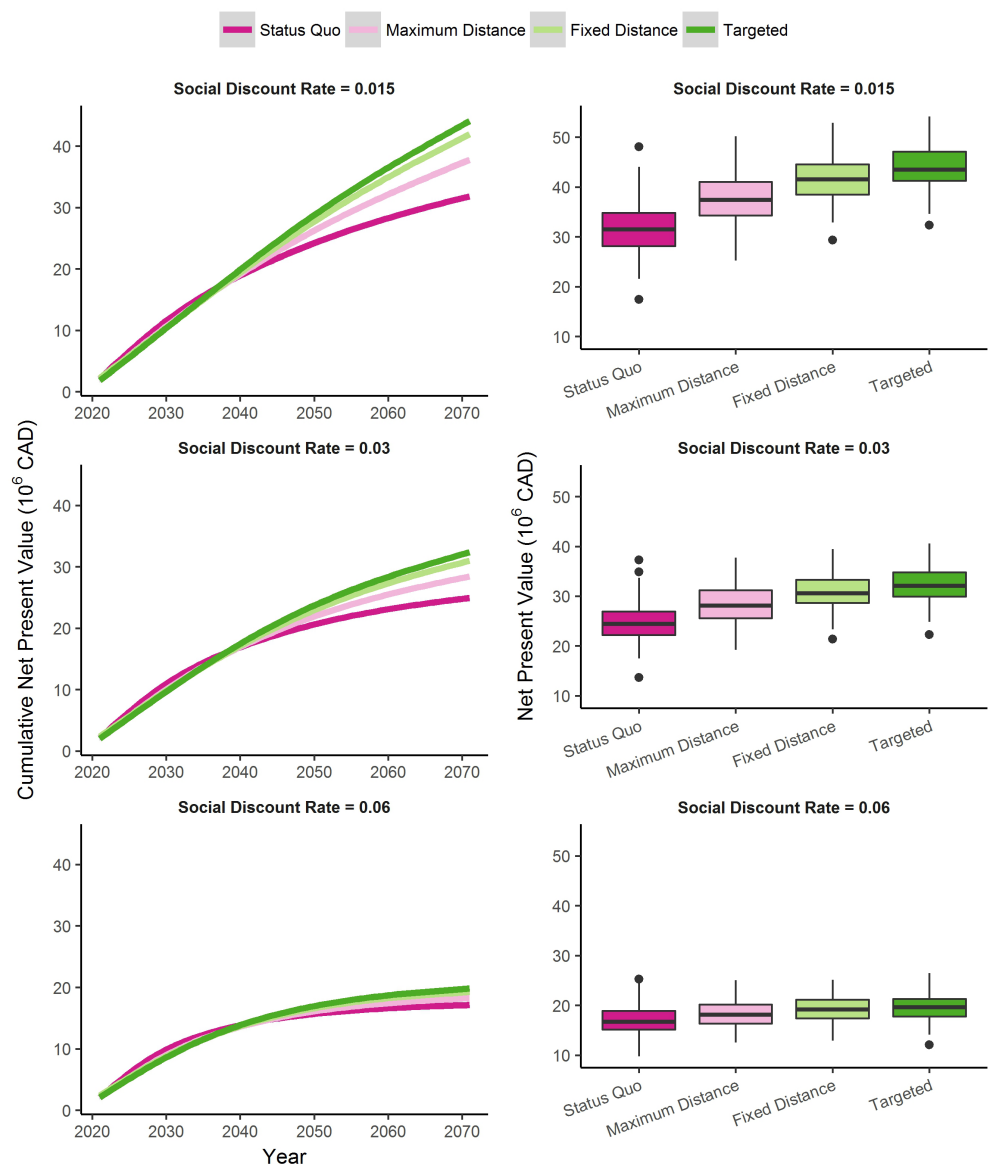
Net present values for each MPA scenario, taking into account three levels of social discount rate. The figures on the left show cumulative net present value, which represents the progression of net present values for each of the years in the time horizon. The solid lines display the mean of all replicates (n=100) and the shaded regions represent standard error. The figures on the right show the final net present value for the full 50 year time horizon. Here, the replicates are represented using a box plot. The median line is surrounded by the 25% and 75% quartiles, whiskers show the limits of 1.5 times the inter-quartile range, and points beyond that range are considered outliers.

In any given year, the differences between management scenarios in terms of biomass, catch, or mean distance from shore are not very large, particularly among scenarios with added MPAs ( Table 1, Figure 3– Figure 5). However, when compounded over multiple decades, these differences have a meaningful effect on economic value ( Figure 6). For the first ~20 y, all scenarios have similar present catch cumulative values ( Figure 6); however, for the remaining 30 y, the Targeted and Maximum Distance scenarios diverge significantly from the Status Quo and Fixed Distance scenarios. For all values of SDR, NPV was highest for the Targeted and lowest for the Status Quo scenarios. The absolute and relative differences in NPVs between Status Quo and treatment scenarios gradually decreased with increasing SDR ( Figure 6; Table 2). With an SDR of 0.015, 0.03, or 0.06, the mean total value of the Status Quo scenario over 51 years is 32, 25, or 17 million CAD respectively while that of the Targeted scenario is 44, 32, or 20 million CAD.

**Table 2.** Error statistics of Net Present Values for the treatment scenarios (Maximum Distance, MD; Fixed Distance, FD; Targeted, TR) relative to Status Quo (SQ), for the three social discount rates (SDR). n = 100 replicates.

SDR	Scenarios	Mean absolute error (10 ^6^ CAD)	Root mean square error (10 ^6^ CAD)	Mean absolute percent error
0.015	SQ vs MD	7.88	9.50	20.12
0.015	SQ vs FD	10.79	12.20	25.19
0.015	SQ vs TR	12.59	13.84	28.24
0.030	SQ vs MD	5.60	6.77	19.08
0.030	SQ vs FD	6.91	8.13	21.73
0.030	SQ vs TR	7.85	9.01	23.89
0.060	SQ vs MD	3.46	4.30	18.69
0.060	SQ vs FD	3.65	4.54	18.49
0.060	SQ vs TR	3.82	4.72	19.04

**Figure 7.**
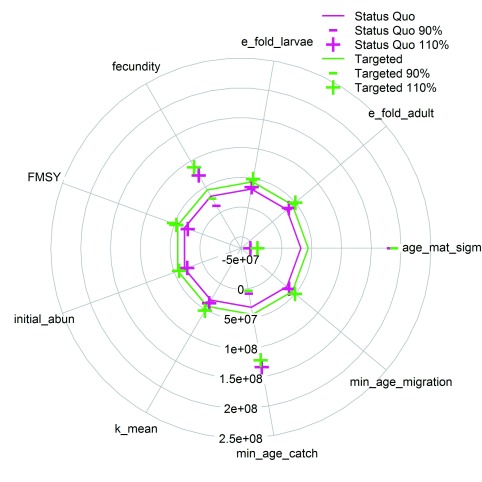
Effect of 10%* increase or decrease of select model parameters on mean final net present value for the full 50 year time horizon with a social discount rate of 0.015. Dotted line indicates the mean value with unaltered parameters. * parameters ‘min_age_migration’ and ‘min_age_catch’ are integer values, therefore sensitivity was determined using ± 1 y. Parameters and their values are defined in Table 3.

Sensitivity analysis revealed that increasing or decreasing the estimates for most parameters did not change the predicted NPV, nor influence the conclusion of the model ( Table 3, Figure 7). NPV increases with an increase in fecundity, but the Targeted scenario still produces more benefit than the Status Quo. The only exception is that when minimum age of catch is increased by one year, the Status Quo scenario produces a higher NPV than does the Targeted scenario.

**Table 3.** Values and definitions of the parameters used in the sensitivity analysis.

Parameter	Definition	Low Value (90%)	Normal Value (100%)	High Value (110%)
*age_mat_sigm*	Sigmoid of the logitic curve for age at sexual maturity. Fish begin maturing at 2 y, 50% at 4 y and all are mature at 6y	3.6	4	4.4
*e_fold_adult*	The e-folding scale for larvae in km (the distance at which there will be fewer settlers by a factor of e)	245	272	300
*e_fold_larvae*	The e-folding scale for larvae in km (the distance at which there will be fewer settlers by a factor of e)	140	156	171
*fecundity*	Size dependent fecundity (eggs per kg of female)	450000	500000	550000
*FMSY*	Fisheries mortality at Maximum Sustainable Yield	0.25	0.28	0.31
*initial_abun*	Number of fish when model is initialized	225000000	250000000	275000000
*k_mean*	Von Bertalanffy growth model parameters - mean growth coefficient (1/year)	0.12	0.13	0.14
*min_age_catch*	Age at which fish can be caught	3	4	5
*min_age_migration*	Age at which fish begin adult migration	5	6	7

## Discussion

These findings suggest that well-designed MPA networks can provide net benefits to the fishing industry, while poorly designed networks can have some deleterious effects, such as an increased probability of moratoriums. This dichotomy highlights the importance of objectively measuring outcomes when evaluating management options. Our R package provides an ideal platform for such studies since it is open source and adaptable. The user can simply input a different set of biological parameters, study area, or fisheries parameters to evaluate similar management scenarios on a different species, or area. Given that spatially explicit population models for multiple species are critical to properly evaluate MPA network design ( Moffitt *et al.*, 2011), we have designed our package so that the biological parameters are easy to modify. At the next level of complexity, the user can create customized functions that create management scenarios or input predefined management scenarios by supplying the necessary shapefiles containing geospatial vector data. Finally, the user can edit whole modules or sub-modules if they wish to explore specific questions such as the effect of non-random dispersal or interactions between species. It is worth noting, however, that adding complexity such as evaluating multiple species at one time would require substantially higher computational power. As a result, simplification in some other areas of the model may be necessary if computational power is a limiting factor.

In terms of network design principles, adding MPAs consistently increased the biomass, but not all MPA networks were equal in terms of Net Present Value. Well-connected MPA network design is thought to reduce population demographic variability by diversifying the potential source populations ( Andrello *et al.*, 2015; Costello & Polasky, 2008; Cowen & Sponaugle, 2009). Correspondingly, our results indicate that abiding to principles of connectivity (Fixed Distance scenarios) and protecting productive habitats (Targeted scenarios) increases the NPV by ensuring well-connected populations. By combining the positive effects of population connectivity, protection of vital habitats, and near-shore source habitats, the Targeted scenario provides the greatest net benefit to the fishing industry.

Intuitively, we expected to see mean fishing distance from shore increase in scenarios with MPAs since we assumed fishermen would be displaced by the presence of MPAs. Our findings were entirely contradictory to these expectations. In all scenarios with MPAs, mean fishing distance from shore decreases relative to the Status Quo since MPAs in our model promote the presence of near-shore source populations that cannot be depleted by fishing. In these scenarios, fishermen spend less on fuel and labour since they do not travel as far. It should be noted here that our model currently assumes that fishermen can have a home port anywhere on the shoreline, which minimizes displacement in our model. In reality, there will be much greater social, economic, and logistical constraints on MPA placement. However, our model has the ability to assess the economic and ecological consequences of any arrangement of MPAs that managers determine to be feasible.

The efficacy of any MPA network is greatly influenced by the level of compliance among commercial fishermen in that region ( McClanahan, 1999). For this reason, we conducted this cost-benefit analysis from the operational point of view (i.e., both the costs and benefits are directly connected to the commercial fishery). As a result, the model outcome is most relevant to the fishermen and can help to make a case for compliance. Mascia (2003) recommended that one measure of performance for an MPA should be how well it enhances the livelihoods of fishermen. Therefore, we should describe the net benefits as they relate to the people most immediately affected by the new MPA network.

A complete evaluation of all costs and benefits associated with MPA networks is beyond the scope of this study. The model does not include other benefits associated with expanding the MPA network. Indeed, benefits are not limited to only increasing fisheries’ profits via increasing yields. Other benefits include job creation related to recreational fisheries or tourism and maintaining other ecosystem services; the value of which exceeds that of the commercial fisheries itself ( Agardi, 1997; Armstrong, 2007; Ghermandi & Nunes, 2013; Lester *et al.*, 2009; Pendleton *et al.*, 2012). In early iterations of the model, we included the costs of establishing and maintaining the MPAs, but the NPV estimates were consistently negative and large. This is to be expected since we are not considering the full suite of benefits listed above. A full treatment of costs and benefits would provide important information for governments seeking to justify the large expense associated with MPAs and would therefore be a rich area for future development.

One trend made clear in our results is that the benefits are realized on long time scales. Fishermen must follow the restrictions for up to 20 years before the net benefits of the Targeted scenario clearly surpass those of the Status Quo. This requires patience and a commitment to preserving a resource for future generations, particularly in the face of inevitable uncertainty in how the cod population will respond along the way. The standard market discount rate tends to be impatient and may not be appropriate for valuing future ecosystem services with high cultural value. If the environmental outcome will affect multiple people and generations, there is a higher social value placed on the service ( Hardisty & Weber, 2009). Therefore, it is justified to apply a lower social discount rate when evaluating future costs and benefits. Social discount rate can have a strong influence on the recommendations from cost-benefit analysis. With increasing discount rates, there is less incentive to preserve fish stocks to generate future profits ( Sanchirico *et al.*, 2006). In our study, the net benefits of the enhanced MPA network scenarios are diminished when SDR >3%. Similarly, Zimmermann *et al.* (2015) found that the difference in NPV between their fishery models was indistinguishable at discount rates >5%.

Sound reserve design has potential for directly enhancing fisheries and preserving sensitive species. Additionally, evidence shows that paying attention to the network design can make populations more resilient to both natural and anthropogenic disturbances. For example, networks can be tailored to better cope with the threats imposed by ongoing climate change ( McLeod *et al.*, 2009). Specifically, a system’s resilience could be enhanced by considering MPAs’ size, shape, risk spreading (i.e., protection of a variety of habitat types), protection of ecologically critical areas, degree of connectivity, maintenance of important functional groups, and external sources of stress like pollution ( Green *et al.*, 2015; McLeod *et al.*, 2009). These aspects can be addressed in the model presented here via some shape of a Targeted scenario, which again lends support to this management strategy.

Our modeling results suggested that the capacity for Atlantic cod populations to significantly recover densities comparable to those observed before the 1960s is limited in every management scenario considered. Similarly, one study reported that only one of 12 stocks of Northwest Atlantic cod analyzed showed ‘substantial recovery’, despite moratoria and fishing quota restrictions established after mid 1990s ( Shelton *et al.*, 2006). Another study by Swain & Chouinard (2008) predicted extinction of cod within the next 20 years (with fishing) or 40 years (without fishing) given the current levels of productivity. As argued by Shelton *et al.* (2006) the low productivity in cod populations documented after the big collapse, as indicated by higher natural mortalities and reduced growth rates ( Swain *et al.*, 2003), might hinder recovery along with the negative impacts of continuing fishery practices. Although our results are consistent with this previous work, our model makes multiple simplifying assumptions about biotic (e.g. ecological interactions) and abiotic (e.g. oceanographic processes) variables that can drive population dynamics and potentially contribute to ecosystem regime shifts. Additionally, our model does not predict any extinctions using the current parameters- possibly because we did not incorporate an Allee effect at low population densities ( Stephens & Sutherland, 1999).

While our model currently provides interesting results regarding MPA design, there are some key aspects to consider when interpreting these findings. This model only considers the interaction between the fishing industry and a single commercial species. This removes possibly important interactions with cod predators ( Cook *et al.*, 2015), competitors ( Minto & Worm, 2012), or prey ( Worm & Myers, 2003). We believe that by choosing a relatively high natural mortality rate that reflects the new ecological reality ( Swain & Chouinard, 2008), we have incorporated the bulk of these ecosystem effects. We have considered both larval and adult dispersal to be entirely random in the model, which neglects potentially important oceanographic, migratory, and homing behaviours ( Green *et al.*, 2014; Lawson & Rose, 2000). Similarly, carrying capacity is randomly generated from a realistic distribution, which ignores the true spatial subtleties of actual cod habitat. However, much of the information needed to incorporate such spatially explicit behaviours and habitats is currently unavailable. Our focus on a single species also eliminates the possibility of a mixed species fishery or evaluating the consequences of switching to alternative species (e.g., crab, and shrimp, and lobster). Although testing multiple species simultaneously was beyond our scope, other studies have employed different techniques for doing so. White *et al.* (2010) calculated unweighted means across species for both recruitment and yield, as well as weighted means that represented the relative commercial landings of each species. Sala *et al.* (2002) incorporated data from seven commercially important species and then used optimization algorithms to choose the most effective combination of MPAs.

In conclusion, the fishing industry stands to benefit financially from well-designed MPA networks (e.g., the Targeted scenario) through increased yields, lower operating costs for the commercial fishermen, and a lower probability of a moratorium. Under scenarios with new MPAs some traditional fishing locations may have been closed, thereby displacing fishing efforts. However, the spill-over effects of the well-designed MPA networks more than compensated for any displacement by providing near-shore source populations and ultimately decreased mean fishing distance from shore. Further, targeted protection of adult habitat can produce long-term financial benefits that exceed those associated with the other simulated MPA network scenarios. These findings demonstrate the power and flexibility of our spatially explicit package in assessing the costs and benefits of different MPA network designs.

## Data and software availability

The tabulated output for the model is available at https://doi.org/10.5281/zenodo.260444

### Software access

TThe R toolkit can be accessed and downloaded at https://github.com/remi-daigle/BESTMPA

### Archived source code as at the time of publication

https://doi.org/10.5281/zenodo.259997

### Software license

Published under the MIT license.

## Supplementary material

**Appendix Figures A1–99.**

Map of eastern Canadian EEZ (Exclusive Economic Zone) and examples of different scenarios for the planning of Marine Protected Areas (MPAs). The “Status Quo” scenario (purple) has all existing MPAs ≥ 400 km ^2^. The MPAs in the “Maximum Distance” scenario (green) have been placed to maximize the distance between them. The MPAs in the “Fixed Distance” scenario (red) have been placed to optimize the distance between them (~75km). The cod’s breeding locations in the “Targeted” scenarios (blue) have been protected by default and optimal distance MPAs have been added exclusively in habitat suitable for cod. Scenarios from replicate 2–100, and replicate 1 is available in Figure 2.

.

Click here to access the data.
