# Supplementary material for: An adaptable toolkit to assess commercial fishery costs and benefits related to marine protected area network design
Source: F1000Res. 2017 Feb 22;4:1234. Originally published 2015 Nov 9. [Version 2] doi: 10.12688/f1000research.7312.2 (PMC5345778; doi:10.12688/f1000research.7312.2)
Supplement: Peer review history

# Peer review: Referee response for version 2

**DOI:** 10.5256/f1000research.11621.r20466

**Published:** 2017-03-03

**Authors:** Marco Andrello

**Affiliations:** 1 Centre of Evolutionary and Functional Ecology, EPHE Laboratory “Biogeography and Ecology of Vertebrates, CEFE UMR 5175, CNRS, University of Montpellier, Montpellier, France

I would like to thank the authors for this excellent revised version and for having addressed all my previous comments. This paper present a very useful toolkit for assessing different spatial scenarios of fisheries management, including the creation of marine protected areas. The proposed case study on Atlantic cod illustrates the potential of the toolkit and the results are discussed thoroughly.

 The sensitivity analysis shows the importance of some parameters for the model results. In particular, it shows that when minimum age of catch is increased by one year, the Status Quo scenario produces a higher NPV than does the Targeted scenario. This seems an important result: if I understand correctly, it could imply that setting a higher age at catch would ensure an optimal management of the fishery and achieve better economic results than creating MPAs according to a targeted scenario. This might discourage further developments and extension of the current MPA system. However, as the authors state in this article, there are multiple benefits associated with the creation of MPAs, such as the ease of implementation relatively to some fishery regulation (would it be possible to set precisely a minimum age of maturation?) and compliance (would it be easy to control that the minimum age is respected?). I think that the final decision process will be complex and beyond the scope of this case-study presentation, for which the authors have already provided an ample discussion.

 Some minor points:

- In the “Population dynamics” section the term “fisherman” is used as singular (twice) where it should be plural (“fishermen”), please check and correct.
- Table 1. It seems that the legend has a mistake for the moratorium part? At least, it should say that “moratorium” is a percent occurrence probability (if I understand correctly)

Thanks for this useful and nice contribution.

I have read this submission. I believe that I have an appropriate level of expertise to confirm that it is of an acceptable scientific standard.

# Peer review: Referee response for version 1

**DOI:** 10.5256/f1000research.7880.r11831

**Published:** 2016-01-25

**Authors:** Marco Andrello

**Affiliations:** 1 Centre of Evolutionary and Functional Ecology, EPHE Laboratory “Biogeography and Ecology of Vertebrates, CEFE UMR 5175, CNRS, University of Montpellier, Montpellier, France

As a referee, I am asked to comment on "whether the work has been well designed, executed and discussed, not whether it is of importance or particular novelty". The toolkit is presented quite clearly. The purpose of this article was also to assess the management of cod. A positive point of this point is the ability to assess the economic and ecological consequences of MPAs spatial planning. I like this point very much.

This article lacks a sensitivity analysis of the model results to all the parameters used in the model. Many parameters are uncertain, so a sensitivity analysis will be helpful. Also, you should not use an ANOVA to examine simulation results (see White *et al*. 2014 ^1^).

There are other points that should be addressed:

- Egg production (0.5 million eggs per kg of female) is missing a reference.
- Is the random walk for larval dispersal and adult movement realistic?
- A beta distribution for larval mortality seems fine; but a normal distribution for adult mortality can give values above 1 or below 0, which is problematic.
- Why was the size distribution of the newly generated MPAs based on that of the World Database of Protected Areas? Existing protected areas are not optimally sized.
- The maximum distance scenario is the best in terms of biomass. Is there a "dramatic" increase in this scenario? Biomass attains more 100 kT in 2043 (the decreases). Compared to biomass in the other scenarios, this increase in relevant; What about the historical biomass?
- It is not clear to me how the profitability ratio works.
- Please define the summary statistics used in Table 1. What are "distance" and "moratorium"? Are values averaged over the fifty years (2000-2050)?

I do not have a sufficient level of expertise to evaluate the Cost-benefit analysis.

I have read this submission. I believe that I have an appropriate level of expertise to confirm that it is of an acceptable scientific standard, however I have significant reservations, as outlined above.

## References

[1] WhiteJRassweilerASamhouriJStierAWhiteC: Ecologists should not use statistical significance tests to interpret simulation model results. *Oikos*.2014;123(4) : 10.1111/j.1600-0706.2013.01073.x 385-388 10.1111/j.1600-0706.2013.01073.x

# Response

**Published:** 2017-02-22

**Authors:** Remi Daigle

**Affiliations:** University of Toronto, Canada

*"As a referee, I am asked to comment on "whether the work has been well designed, executed and discussed, not whether it is of importance or particular novelty". The toolkit is presented quite clearly. The purpose of this article was also to assess the management of cod. A positive point of this point is the ability to assess the economic and ecological consequences of MPAs spatial planning. I like this point very much.*

*This article lacks a sensitivity analysis of the model results to all the parameters used in the model. Many parameters are uncertain, so a sensitivity analysis will be helpful. Also, you should not use an ANOVA to examine simulation results (see White et al. 2014 1)."*

- **Sensitivity Analysis: We agree that, in light of parameter estimate uncertainty, sensitivity analysis was an important addition to this study.  We modified select parameters by ± 10% (or ± 1 y for integer values) for 25 replicate model runs and then compared the mean final net present value for the full 50 year time horizon to that of the full model (n = 100) in order to quantify influence of that parameter estimate on model output.  We did this using the social discount rate of 0.015 because this is the value for which differences among the scenarios are most pronounced.  We also focused only on the Targeted and Status Quo scenarios because they are the most extreme scenarios.  We discovered that the most sensitive parameters are minimum age of catch and fecundity.  **

- **Use of ANOVA:  We removed any reference to the ANOVA. Instead we report Error statistics (mean absolute error, Root mean error, and mean absolute percent error)**

*"There are other points that should be addressed:"*

*- Egg production (0.5 million eggs per kg of female) is missing a reference.*

- **We included a new reference (Otterå, 2004) to support this parameter value.**

** **


**-** *"Is the random walk for larval dispersal and adult movement realistic?"*

- **Not quite, it does not really respect possible home ranges (adherence to an area), but it is scaled to real movement patterns. The random walk is the simplest approximation, and we make it possible for the users to use the best available connectivity information. This could be data from tagging, bio-physical models, or in the absence of more accurate data, the user can simply use a random walk. **

*- "A beta distribution for larval mortality seems fine; but a normal distribution for adult mortality can give values above 1 or below 0, which is problematic."*

- **We have added a step that eliminates any values above 1 or below 0 (M <- M[M<=1&M>=0]) which given the cod parameters was exceedingly rare.**
**  **

*- "Why was the size distribution of the newly generated MPAs based on that of the World Database of Protected Areas? Existing protected areas are not optimally sized."*

- **Andrello brings up an excellent point that the existing MPAs are not optimally sized. However, our intent of drawing from the existing size distribution was to work within the bounds set by previous management decisions. Our assumption here is that decision makers would consider the size of the MPA selected as reasonable based on precedent set in other areas. We revised the text at the end of the “Management strategies scenarios” section to reflect our rationale.  **
**  **

*- "The maximum distance scenario is the best in terms of biomass. Is there a "dramatic" increase in this scenario? Biomass attains more 100 kT in 2043 (the decreases). Compared to biomass in the other scenarios, this increase in relevant; What about the historical biomass?"*

- **We had problems with the “virtual_fish_ratio” of the original model, where 1 ‘virtual’ fish represented 20000 real fish. This led to unnecessary variability in the results. The model has been redesigned and the “virtual_fish_ratio” has been eliminated making the new version a truly individual based model. The biomass for the maximum distance scenario is not as high with the new model.**
**  **

*- "It is not clear to me how the profitability ratio works."*

- **We revised the description to clarify how the profitability ratio was calculated (see first paragraph of the “Management strategies scenario” section.  In the process, we discovered we had neglected to include one of the necessary citations, which is now added to the the References section.  **
**  **

*- "Please define the summary statistics used in Table 1. What are "distance" and "moratorium"? Are values averaged over the fifty years (2000-2050)?"*

- **We have edited the table legend to address these questions.**

# Peer review: Referee response for version 1

**DOI:** 10.5256/f1000research.7880.r11198

**Published:** 2015-12-03

**Authors:** Pedro Peres-Neto

**Affiliations:** 1 Department of Biological Sciences, Université du Québec à Montréal, Montréal, Québec, Canada

This paper reports a quantitative tool that can be used to analyze the economical impact of different network designs for marine protected areas. The framework is quite flexible and will provide a useful toolkit for discussing the pros and cons of different designs. That said, I feel that it considered very little discussion regarding other potential algorithms and also how to consider multiple species at the same time. In particular, would the authors suggest to model different species separately and then build a consensus across different MPA designs? Some discussion on this issue would be particularly important. Another issue is environmental heterogeneity. I think it would be important that the authors at least discuss how environmental heterogeneity should be taken into account in designing MPA across different scenarios. Large habitat heterogeneity within a given area or across areas would certainly lead to different design decisions than small heterogeneity. How does the matrix and quality of corridors should be considered. In sum, I think this is a much needed tool but the authors need to provide some additional information on how they see it being applied considering multiple species and environmental heterogeneity.

I have read this submission. I believe that I have an appropriate level of expertise to confirm that it is of an acceptable scientific standard.

# Response

**Published:** 2017-02-22

**Authors:** Remi Daigle

**Affiliations:** University of Toronto, Canada

- In response to multiple species: The modular design of the tool kit could allow users to add this level of complexity if so desired, but the current structure would require higher computational power to run a multiple species model. We address this at the end of the first discussion paragraph.
- Although we are not evaluating multiple species in this paper, we provide some citations and descriptions of approaches tried by other researchers. (See Discussion Paragraph 9.)
- We have modified the model so that habitat carrying capacity is now a customizable input. While our paper does not focus on the issue of habitat heterogeneity, the BESTMPA package could now be used to investigate this question.
